# Crowdsourcing Novel Childhood Predictors of Adult Obesity

**DOI:** 10.1371/journal.pone.0087756

**Published:** 2014-02-05

**Authors:** Kirsten E. Bevelander, Kirsikka Kaipainen, Robert Swain, Simone Dohle, Josh C. Bongard, Paul D. H. Hines, Brian Wansink

**Affiliations:** 1 Department of Communication Science, University of Amsterdam, Amsterdam, the Netherlands; 2 VTT Technical Research Centre of Finland, Tampere, Finland; 3 College of Engineering and Mathematical Sciences, University of Vermont, Burlington, Vermont, United States of America; 4 Institute for Environmental Decisions, Consumer Behavior, ETH Zurich, Zurich, Switzerland; 5 Charles H. Dyson School of Applied Economics and Management, Cornell University, Ithaca, New York, United States of America; University of Tolima, Colombia

## Abstract

Effective and simple screening tools are needed to detect behaviors that are established early in life and have a significant influence on weight gain later in life. Crowdsourcing could be a novel and potentially useful tool to assess childhood predictors of adult obesity. This exploratory study examined whether crowdsourcing could generate well-documented predictors in obesity research and, moreover, whether new directions for future research could be uncovered. Participants were recruited through social media to a question-generation website, on which they answered questions and were able to pose new questions that they thought could predict obesity. During the two weeks of data collection, 532 participants (62% female; age  =  26.5±6.7; BMI  =  29.0±7.0) registered on the website and suggested a total of 56 unique questions. Nineteen of these questions correlated with body mass index (BMI) and covered several themes identified by prior research, such as parenting styles and healthy lifestyle. More importantly, participants were able to identify potential determinants that were related to a lower BMI, but have not been the subject of extensive research, such as parents packing their children’s lunch to school or talking to them about nutrition. The findings indicate that crowdsourcing can reproduce already existing hypotheses and also generate ideas that are less well documented. The crowdsourced predictors discovered in this study emphasize the importance of family interventions to fight obesity. The questions generated by participants also suggest new ways to express known predictors.

## Introduction

The continuous rise in the prevalence of obesity is evident throughout the world [1]–[3]. In the United States in 2010, the rate of obesity was 16.9% among children and adolescents [4] and 35.7% among adults [2]. Globally, the prevalence of obesity was 9.8% in men and 13.8% in women in 2008 and estimated to be increasing in most regions of the world [3]. Alarmingly, weight-related health problems such as diabetes and cardiovascular diseases, which formerly have not emerged until adulthood, are now being diagnosed in children [5], [6]. As the rate of pediatric obesity increases and has long lasting effects during adolescence and adulthood, childhood is the crucial time for prevention.

In the past decades, a multitude of factors that play an important role in the development of obesity have been examined by means of various research methods and designs. The majority of studies can be classified as expert driven; that is, experts or professionals test hypotheses by posing (validated) questions that are often based on existing literature within their domain. However, it is possible that there are determinants which experts have left untouched. The current study presents ‘crowdsourcing’ as an innovative bottom-up approach to detect possible unexpected or new predictors of obesity by using the knowledge of the general (non-expert) public.

Web-based crowdsourcing is a rather anonymous, fast, and inexpensive method to generate new hypotheses and discover unexpected issues which might have been overlooked by professionals [7]. A recent study suggests that causal factors of behavioral outcomes can be discovered by means of crowdsourcing, for example, people’s body mass index [8]. To date, the generation of new insights and ideas through crowdsourcing has been under increasing attention for commercial use [9], [10]. Research has shown that a crowdsourcing process can generate more novel ideas than professionals [10]. In the present study, the process of crowdsourcing to discover (new) childhood predictors of obesity happened as follows. Participants were recruited through social media to a website on which they were asked to provide their current weight and height and answer questions about their experiences and behaviors during their childhood that could be predictive of their current body mass index (BMI). Notably, after answering the questions the participants were the ones who created new questions that were then answered by other participants. The web site predicted their BMI based on the growing data set. Hence, investigating possible early markers for obesity was outsourced to a non-expert community. Collectively, these non-experts could uncover already identified as well as unexpected childhood determinants of obesity [8].

Understanding the early causes of weight gain has been the focus of a vast amount of research and many determinants of overweight and obesity have been identified [11], [12]. Few studies have been conducted by means of recalled childhood determinants of later adult weight status. As parents play an important role in shaping children’s food habits, previous recall studies have shown a particular relation between adult eating habits and parenting and feeding styles experienced during childhood; that is, rules which restrict or encourage food intake, or rules where food is used to reward or punish behavior [13]–[16]. Although longitudinal research is warranted, evidence exists that parental feeding styles such as a restrictive feeding style or controlling what, when, and how much the children eat (i.e., authoritarian/demanding or adult-controlled feeding style) are related to higher BMI later on [17]. It is argued that the amount and style at which parents exert power over their children have an influence on the children’s self-control [14]. The parenting style in which parents use a cooperative feeding style and share the responsibility of food intake with their children (i.e., authoritative/responsive style) has been recommended [18]. In addition, general parenting styles in which parents are uninvolved and low in warmth and caring, or low in structure and support are associated to a higher weight later on in life [14], [19].

Dietary intake and physical inactivity have been identified as the two major contributing lifestyle factors to overweight and obesity [20]. For example, correlational as well as longitudinal studies have shown that skipping breakfast, consumption of non-home cooked meals, an increased soda consumption and high-fat food intake are related to overweight and obesity [20]–[25]. Watching television (TV) or playing computer games have been shown to contribute to physical inactivity and increased sedentary behavior [26]–[28]. An additional predictor for a (un)healthy lifestyle that is associated with an increased weight is a shortage of hours of sleep [29].

The built environment has also been found to contribute to people’s physical activity and dietary patterns. For instance, pavements or access to recreational facilitates have been associated with a higher level of children’s physical activity whereas the local food environment (e.g., convenience store or (fast-food) restaurant density) has an impact on people’s food intake [30], [31]. Nevertheless, the social and built environment where people grow up in is largely dependent on their socioeconomic status (SES) and educational level [22], [32]. A lower SES and educational level during childhood has been consistently found to be related to a higher BMI later on in life [33], [34]. As energy-dense foods are relatively low in cost, low-income households are more likely to have low quality diets (e.g., low fruit and vegetables consumption) [34].

Furthermore, several studies have examined the effect of psychosocial factors on the origin of overweight and obesity. For example, low social acceptability and low psychological well-being (e.g., negative emotions [33], low self-esteem [35], and depression [36], [37]) have been found to contribute to a higher BMI later on in life. Finally, although behavioral and environmental factors have been shown to determine overweight and obesity, biological factors should not be discarded as literature has shown a child to adult adiposity relationship and biological predispositions to weight gain [33], [38]

The research mentioned above only provides a brief summary of what might potentially be regarded as the most obvious childhood predictors of obesity by the participants in the crowdsourcing process. As there is a need for effective and simple screening tools for evaluating overall lifestyle quality and associating it with obesity development, the present study had two goals. The first goal was to examine whether it is possible for a non-expert community to identify known childhood predictors of obesity, using a crowdsourcing process. The second and more important goal was to find out whether crowdsourcing can be used as a low-effort method to discover potential new childhood determinants of adult obesity. In summary, the study explored the feasibility of crowdsourcing as a method to assess determinants of obesity.

## Methods

### Ethics Statement

The study was approved by the Institutional Review Board of the University of Vermont. All participants received information about the study and study procedures upon entering the crowdsourcing website, after which they were required to give their informed consent online before entering the study.

### Crowdsourcing Procedure

**Figure 1:**
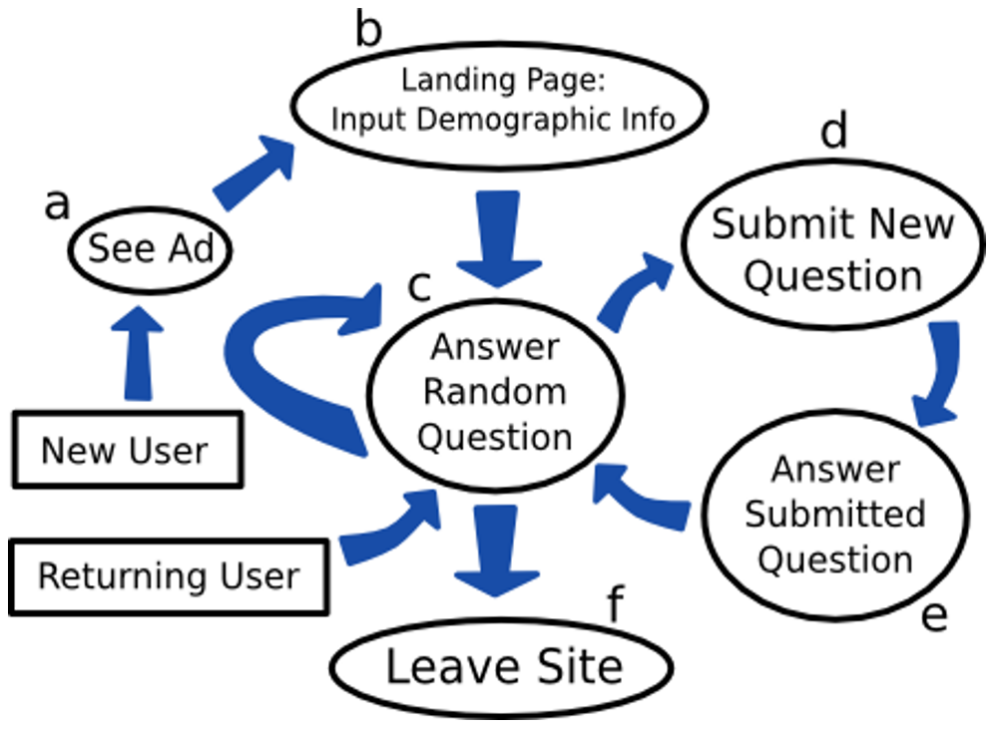
Flow chart of a user's interaction with the crowdsourcing website.

Figure 1 illustrates the crowdsourcing process. Participants were recruited (Figure 1a) through posted notices on reddit.com, which is a user-generated content news site. Notices were posted on specific sections focused on dieting (www.reddit.com/r/keto), weight loss (www.reddit.com/r/loseit), and parenting (www.reddit.com/r/parenting). Reddit.com and the specific sections were chosen as the initial recruitment channel because the users could be expected to be interested and motivated in participating in a study that might help them improve their lifestyle and that involves user-generated questions.

**Figure 2:**
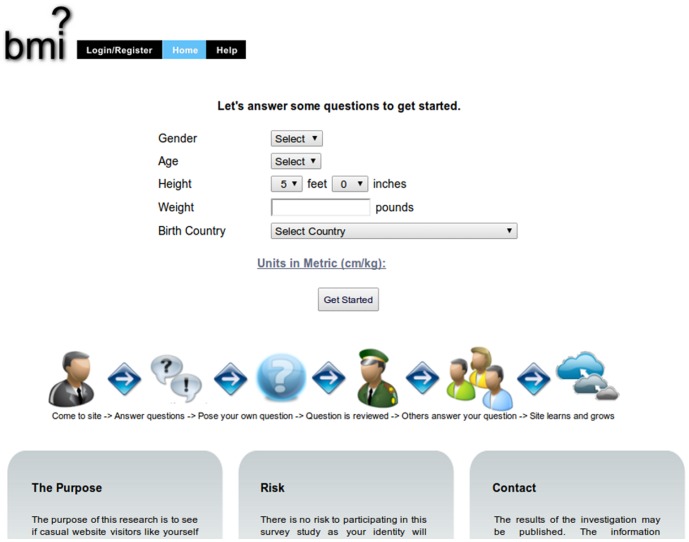
Screenshot showing the landing page of the crowdsourcing website.

**Figure 3:**
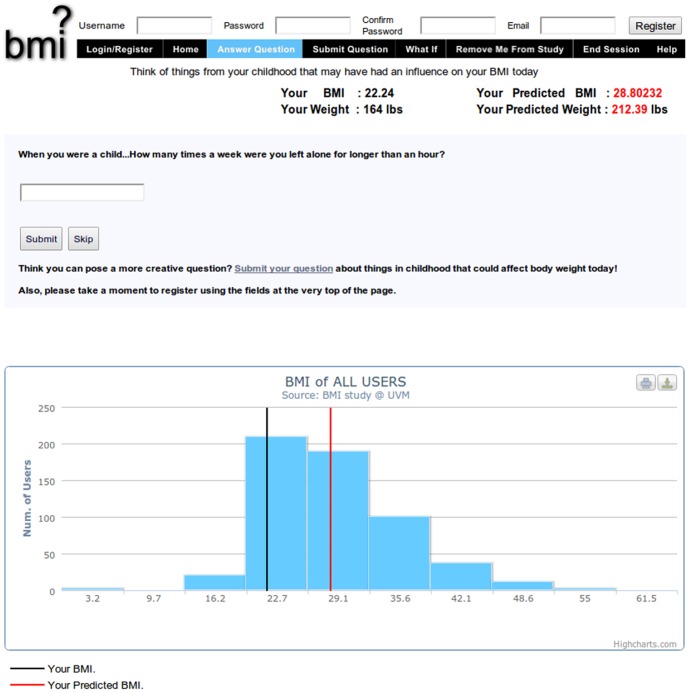
Screenshot showing a question page on the crowdsourcing website.

The website that was used in this study for crowdsourcing was based on a prior experiment [8] and modified to collect crowd-suggested childhood predictors of adult BMI. As seen in Figure 2, participants who visited the site were at first asked to input their age, gender, weight, height, and birth country as background information (Figure 1b). The participants could choose whether to fill in their weight and height in kilograms and centimeters or pounds and inches. After entering this information, they were directed to answer questions found on the site (Figure 1c). Within the survey, a participant’s actual BMI was displayed alongside their predicted BMI, which was updated each time the participant answered a question. The participant’s actual and predicted BMI were superimposed over a histogram which displayed the distribution of all participants’ BMIs (see Figure 3). Predicted BMI was calculated by performing linear regression on all of the questions and responses provided by previous visitors to the site, supplying the current subset of responses provided by the current user to the resulting model, and displaying the prediction of the linear model on the website as `predicted BMI’. The site was initialized by ‘seeding’ it with questions that the investigators expected would correlate with BMI. These seed questions were: “When I was a child, I was bullied”, “When you were a child, did you own a bike?”, and “When you were a child, how many times a week did you eat at a fast food restaurant?”

**Figure 4:**
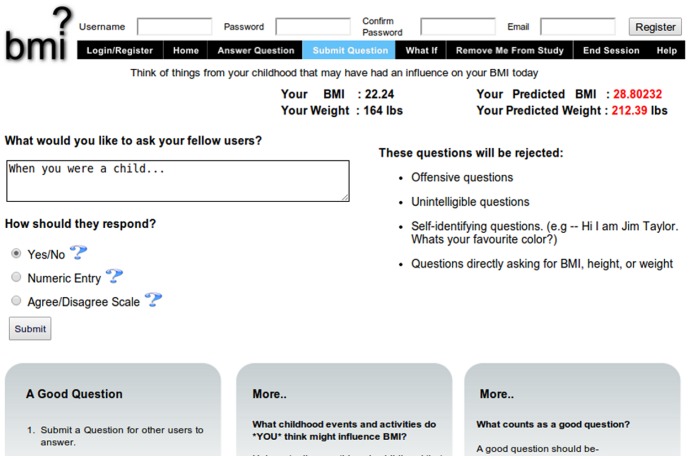
Screenshot showing the new question submittal page on the crowdsourcing website.

At any time, users could pose their own questions (Figure 1d-e). As shown in Figure 4, the site allowed users to pose questions with three types of responses: yes/no, a disagree/agree rating on a 1-7 Likert scale, or a number. The users were provided with a suggestion for how to begin the question (“when you were a child…”) in order to constrain them to asking questions about childhood behavior. Questions posed by users were sent to be approved by the moderator and added to the website if deemed suitable. A question was determined to be unsuitable if it met one or more of the following exclusion criteria: the user self-identified themselves (e.g., “I’m John Smith and would like to know if…”); the user posed a question likely to be nearly perfectly correlated with BMI (e.g., “What is your BMI?”); the user posed a question with offensive language; or the user posed a question likely to upset other users. Once a question was approved by the moderator, the question would immediately be added into the survey, after which it would be seen by subsequent users visiting the site.

Data were collected during a pre-defined period of two weeks: from 8 – 23 November 2012. There was no predetermined target sample size because the survey was voluntary and it was not possible to predict how many people would participate or how many questions and answers the crowd would generate. Nevertheless, rough indicators of expected sample size can be collected from prior work. Previous studies on crowdsourcing in relation to residential electric energy consumption and body mass index instantiation have had relatively small sample sizes (N = 58 and N = 64) with a recruitment period of 6 days up to 3 months, respectively [8]. Another example is a crowdsourcing contest for sustainable design which had a larger sample size (N = 1,233) with submitted 605 designs and 3,594 evaluations of these designs over two months [39]. For the current explorative study, a fixed time period of two weeks was set beforehand and the final sample size was the number of people who participated during this period.

Questions and answers that had been generated by the participants during the two weeks were extracted from the website for analysis. Visitors who gave their background information but did not provide any responses to questions or whose BMI data was missing were excluded from analyses.

## Measures

**Weight status (BMI).** The body mass index (BMI) for each participant was calculated using the established formulas: weight [kg]/height^2^ [m], or weight [lbs]/height^2^ [in] * 703.07, depending on which measurement units the participant chose to use.

**Categorization of crowd-generated questions.** The questions that were generated by the participants were placed into several pre-defined top-level categories (e.g., parenting (feeding) style, healthy lifestyle, home environment, and psychosocial well-being) based on existing research or using a keyword appearance approach. If possible, top-level category questions were further divided into second-level categories. Questions were placed into the ‘healthy lifestyle’ category if they were related to topics identified in research such as diet, physical activity, sleep, watching TV, dental care, or contained the words ‘eat’, ‘drink’, any references to specific food products (e.g. `skim milk’), or the noun or verb forms of `sleep’ or ‘TV’ [11], [21], [28], [29].

A question was placed into the category `home environment’ and further categorized as ‘socioeconomic status,’ ‘parental feeding style,’ ‘parental dieting’ or ‘parenting style’ if it resembled topics that were identified by existing research (e.g., Child Feeding Questionnaire (CFQ) [40], Dutch Eating Behavior Questionnaire for Children (DEBQ-C) [41] or parental dieting or encouragement [42]) and/or contained the noun or verb form of the words ‘poverty,’ ‘punish’ or ‘reward’, or the word ‘parent’, ‘parents’, ‘mother’ or ‘father’ [16], [18], [19], [32], [43]. The remaining questions were categorized by concepts or words that were related to the built environment [30], [31], psychosocial well-being [35], [44], and familial and biological factors [45]. Questions that were ambiguous were ultimately placed in categories based on authors’ intuition. We acknowledge that several questions could be categorized differently (e.g., growing own food might be a marker of a healthy lifestyle or socioeconomic status).

### Strategy of analysis

Participants were divided into weight categories (underweight, normal-weight, overweight, obese) based on their BMI. The characteristics of participants were described by computing the mean for continuous variables (age, BMI) and proportions for categorical variables (gender, birth country).

Associations between the crowd-generated questions and BMI were assessed by calculating correlations between participants’ BMIs and their answers to the questions. Spearman correlations were calculated for categorical variables (no/yes questions) and Pearson correlations for ordinal (disagree/agree scale) and numerical questions. Second, crowd-generated questions were placed into the pre-defined categories and compared with existing literature in order to assess their degree of novelty in comparison to existing constructs or operationalizations of potential predictors of obesity. Finally, questions which were significantly associated with participants’ BMI and assessed to be less well documented in research were correlated with other significant crowd-generated correlates of BMI to explore how they were interrelated. The purpose was to identify behaviors or factors that might co-occur and together give indications of what could explain differences in BMI. The three strongest and the two weakest correlating questions were explored in this manner.

Additional analyses were performed to explore possible interrelationships among conceptually related items and to clarify the relative importance of various correlates. Data was scaled by both the mean and standard deviation. Multivariate analysis was performed using linear regression and exploratory factor analyses. Questions with more than 50% missing values were excluded from the multivariate analysis. The resulting subset consisted of the first 15 questions for all of the 556 participants. The remaining missing values within this subset were filled using multiple imputation [46]. An aggregate linear model was produced from the 10 imputed datasets [47]. Exploratory factor analysis was performed on the first 15 questions with mean-filled missing values. A scree plot analysis and the Kaiser criterion were used as guidelines for the range of factors to investigate. Interpretability criteria were that at least 3 items had significant loadings (>.30) and that the variables that loaded on a factor shared conceptual meaning. In addition, the variables that loaded on different factors had to measure different constructs with higher loadings on one factor than the other.

## Results

The website attracted 556 visitors who provided their background information. After excluding visitors with missing BMI data (*n* = 3, shown in Figure 2) or responses to questions (*n* = 21), the final sample consisted of 532 participants. The mean BMI of the final sample was 29.0, mean age was 26.5 years, 62% were female, and the majority (73%) had been born in the United States. **Table 1** presents the characteristics of participants.

**Table 1:** Participant characteristics.

	All	Underweight(BMI <18.5)	Normal weight (BMI 18.5–25)	Overweight (BMI 25–30)	Obese (BMI >30)
n	532	9	169	155	199
BMI, mean (SD)	29.0 (7.02)	17.4 (0.76)	22.5 (1.71)	27.4 (1.44)	36.2 (5.63)
Age, mean (SD)	26.5 (6.71)	22.9 (4.78)	25.3 (6.22)	25.9 (6.16)	28.3 (7.22)
Female	62%	89%	69%	65%	53%
Birth country					
United States	73%	78%	68%	76%	75%
Canada	9%	11%	10%	8%	9%
United Kingdom	4%	0%	4%	3%	4%
Australia	3%	0%	2%	4%	4%
Other	12%	11%	17%	9%	9%

In addition to the three ‘seed questions’ supplied by the researchers, 35 (7%) of the participants proposed in total 56 new questions. In total, participants provided 10,858 responses to the 59 questions. Out of the total 59 questions that were posed by the participants and seeded by the researchers, 16 questions were significantly correlated (*p* <.05) and 3 questions were marginally correlated (*p* <.10) with BMI (see Appendix S1 for a list of all questions and their correlations with participant BMI).

**Table 2** presents a list of questions that were significantly related to BMI in the order of magnitude of the correlations. It shows that whether someone packed their child a lunch for school, whether meals were prepared with fresh ingredients, whether parents talked about nutrition, and whether the child engaged with their family in regular outdoor activities were strongly related to having a lower BMI later on in life. Family history (e.g., weight of parents and grandparents) and whether food was used as a punishment were related to a higher BMI later on in life. The two weakest significant predictors appeared to be the child preparing his/her own meals more often than parents and being bullied.

**Table 2:** Questions with highest correlations with BMI.

#	Question	Correlation	P value
q53	When you were a child, did someone consistently pack a lunch for you to take to school?	–.345	<.001
q34	When you were a child...did your family primarily prepare meals using fresh ingredients?	–.316	<.001
q59	When you were a child...did your parents talk about nutrition?	–.309	.001
q19	When you were a child... How many times per week did you bring your lunch to school?	–.234	.012
q17	When you were a child did you engage in regular outdoor activity, like hiking or biking, with your family?	–.230	.008
q39	When you were a child, was the food used as a punishment in any ways?	.219	.021
q4	When you were a child, were your parents obese?	.218	<.001
q54	When you were a child...was your maternal grandmother obese	.208	.032
q41	When you were a child...were your grandparents overweight?	.198	.036
q18	When you were a child...How much sleep did you get on an average school weekday?	–.172	.034
q5	When you were a child, did you live in poverty?	.171	<.001
q12	When you were a child, did you drink juice or soda more often than water?	.166	.001
q7	When you were a child, did your parents restrict your food intake?	.155	.002
q6	When you were a child, were you rewarded with food?	.141	.005
q13	When you were a child, did you prepare your own meals more often than your parents did for you?	.130	.012
q1	When I was a child, I was bullied.	.128	.009
q43	When you were a child...Did you have many friends?	–.168	.070
q47	When you were a child... at what age was your first tooth filling?	.179	.081
q25	When you were a child... did your household serve reduced-fat alternatives to traditional foods (e.g. skim milk instead of whole, egg beaters instead of whole eggs, etc.)?	.161	.091

### Crowd-generated questions

The significant and insignificant correlates are shown in **Table 3** under the pre-defined categories of home environment, psychosocial well-being, healthy lifestyle, and family history and biological factors. The categories with the largest number of questions were home environment and healthy lifestyle. The participants identified predictors which are related to a healthy lifestyle such as dietary intake (e.g., whether the family primarily prepared meals using fresh ingredients, *r_s_* = –3.16, *p* <.001, and whether children drank juice or soda instead of water, *r_s_*  = .17, *p*  = .001), physical activity with the family (*r_s_* = –.23, *p*  = .008), hours of sleep (*r* = –1.17, *p*  = .034) and dental care (*r*  = .18, *p*  = .081). Participants also came up with constructs that are topics of attention in research but were not significantly correlated, such as playing outdoors, television watching and several dietary questions related to eating at (fast food) restaurants or at home, (midnight) snacks (*p* >.10).

**Table 3:** What crowd-suggested childhood markers for adult BMI are significant?^1^

Category	Subcategory	Significant	Non-significant
***Home environment***	**Food education**	* (–) Parents talking about nutrition (q59)	Being taught how to cook (q36)
	**Parenting/Parental feeding style**	(+) Food used as reward (q6)	Parents encouraging to clean the plate (q37)
		(+) Food used as a punishment (q39)	Parents prohibiting certain foods (e.g., sweets, sodas) (q45)
		(+) Parents restricting food intake (q7)	Parents allowing to eat whatever you wanted (q40)
		* (+) Preparing own meals more often than parents did (q13)	Parents frequently asking what you were eating (q31)
		* (–) Someone packing a lunch for you to take to school (q53)	Sugary foods being special treat rather than in regular diet (q46)
		* (–) Times per week for bringing your lunch to school (q19)	
	**Parental dieting**	(+) Household serving reduced-fat alternatives to traditional foods (q25) *(marginally significant)*	Mother being constantly on a diet (q21)
			Amount of exercise parents a week (q38)
			Weight/body image being a topic of conversation or concern to the adults in your life (q26)
	**Household status and SES**	(+) Living in poverty (q5)	Being raised by a single mother (q24)
			Parents divorcing (q44)
			Parents having a good healthy relationship (q11)
			Usually eating together with family (q30)
***Psychosocial well-being***		* (+) Being bullied (q1)	Frequency of being left alone for longer than an hour (q20)
		* (–) Having many friends (q43) *(marginally significant)*	Experiencing event causing emotional trauma (q28)
			Facing identity issues which affected you psychologically (q58)
***Healthy lifestyle***	**Diet**	* (–) Family primarily preparing meals using fresh ingredients (q34)	Eating sweetened cereal (q27)
		* (+) Drinking juice or soda more often than water (q12)	Eating candy (q16)
		(+) Household serving reduced-fat alternatives to traditional foods (q25) *(marginally significant)*	Drinking skim milk more often than whole milk (q23)
			Eating between meals (q15)
			Eating late at night (q10)
			Eating home-cooked meals (q29)
			Eating at fast food restaurants (q3)
			Eating at non-fast-food restaurants (q8)
			Family growing their own food (q52)
	**Physical activity**	(–) Engaging in regular outdoor activity with family (q17)	Hours per week playing outdoors (q42)
			Being involved in any competitive sports (q9)
			Catch and other active/outdoor games being your favorite (q33)
			Spending more time playing outdoors than indoors (q51)
			Owning a bike (q2)
	**Sleep**	(–) Hours of sleep on an average school weekday (q18)	
	**Watch TV**		Watching TV while eating dinner (q50)
			Having a meal while watching television (q56)
	**Dental Care**	* (–) Age of first tooth filling (q47) *(marginally significant)*	
***Built environment***			Fast food restaurant within walking distance/a short bike ride (q49)
			Raised on a coast of the United States (q35)
***Family history & biological factors***		(+) Parents obese (q4)	Having any metabolic disorders (q55)
		* (+) Maternal grandmother obese (q54)	Birth weight (q32)
		(+) Grandparents overweight (q41)	

1 For the list of all original questions and their correlations with BMI, see Appendix S1.

* New dimensions for (existing) constructs or operationalizations of potential predictors of obesity.

Using food to reward (*r_s_*  = .14, *p*  = .005) or punish (*r_s_*  = .22, *p*  = .021) behavior as well as restricting food intake (*r*_s_  = .16, *p*  = .02) were associated with a higher BMI. Parents talking about nutrition was associated with a lower BMI (*r*_s_ = –.31, *p*  = .001) as well as having someone pack the child’s school lunch (*r_s_* = –.345, *p* <.001). Interestingly, a well-documented construct about whether children were encouraged to clean their plate was not correlated significantly with BMI among this sample, and neither were several other questions related to restriction (*p* >.10).

Apart from lifestyle and the home environment, predictors that influenced participants higher or lower BMI were related to their psychosocial well-being, such as being bullied (*r_s_*  = .128, *p*  = .009) and having friends (*r_s_* = –.168, *p*  = .07), respectively. In addition, the weight of ancestors were positively correlated to participant’s BMI later on in life but not birth weight or being born prematurely (*p* >.10). Questions related to the built environment were scarce and they were not correlated to participant’s BMI (*p* >.10). “Being bullied” (q1) was the only seed question posed by the researchers that was significantly correlated.

### Interrelated constructs

Ten of the questions could be viewed as either new or under-researched operationalization of an existing constructs or as a novel new potential predictors of obesity. Interestingly, three of the strongest predictors (see q53, q34, and q59 in Table 2) appeared to be the constructs that were also less well documented by research. Therefore, these were closely examined to determine which other significant predictors were correlated with them to identify co-occurring factors. Additionally, the two weakest (significant) predictors were explored (i.e., preparing own meals more often than parents and being bullied). **Table 4** presents the correlations between questions. Interestingly, the constructs in which parents ‘pack lunch,’ ‘prepare meals using fresh ingredients,’ and ‘talk about nutrition’ all show positive correlations in relation to parenting style and a healthy diet and lifestyle (e.g., outdoor activities and sleep). These constructs might indicate a supportive home environment. Talking about nutrition was also correlated with restrictive parenting which might be related to talking about food while restricting children to food.

**Table 4:** Correlates for Five New Interesting Predictors.

		Someone packing a lunch for you to school (q53)	Family preparing meals using fresh ingredients (q34)	Parents talking about nutrition (q59)	Preparing own meals more often than parents did (q13)	Being bullied (q1)
**Home environment**						
q53	Someone packing a lunch for you to take to school	1.000	.223*	.343^**^	–.235*	–.095
q59	Parents talking about nutrition	.343^**^	.338^**^	1.000	–.162	–.133
q13	Preparing own meals more often than parents did	–.235*	–.345^**^	–.162	1.000	.101
q7	Parents restricted your food intake	.060	–.087	.296^**^	.036	.038
q39	Food used as a punishment in any ways	–.104	–.090	.187	.232*	–.013
q6	Rewarded with food	.027	–.113	–.082	.027	.087
q5	Living in poverty	–.086	–.139	–.126	.185^**^	.217^**^
**Psychosocial well-being**						
q43	Having many friends	.249*	.415^**^	.127	–.343^**^	–.345^**^
q1	Being bullied	–.095	–.012	–.133	.101	1.000
**Healthy lifestyle**						
q17	Engaging in regular outdoor activity with family	.394^**^	.276^**^	.292^**^	–.278^**^	–.299^**^
q18	Sleep on an average school weekday	.231*	.234*	.254*	.019	.010
q12	Drank juice or soda more often than water	–.303^**^	–.323^**^	–.414^**^	.089	.165^**^
q25	Household served reduced-fat alternatives to traditional foods (e.g. skim milk instead of whole, egg beaters instead of whole eggs, etc.)	.065	–.017	.086	–.023	–.092
q19	Times per week bringing lunch to school	.763^**^	.249*	.361^**^	–.256*	–.167
q34	Family preparing meals using fresh ingredients	.223*	1.000	.338^**^	–.345^**^	–.012
**Family history & biological factors**						
q4	Parents obese	–.031	–.347^**^	–.118	.096	.174^**^
q41	Grandparents overweight	–.039	–.162	–.234*	.044	.117
q54	Maternal grandmother obese	–.115	–.177	–.139	.122	.130

* p <.05, ** p <.01, *** p <.001.

Notably, preparing meals more often than parents showed negative correlations within parenting style and a healthy diet and lifestyle. It also showed a positive correlation with socioeconomic status (poverty). This indicates that children who prepared their own meals also lived in poverty, had a less healthy lifestyle, and had less support from parents. Not surprisingly, people who were bullied had fewer friends. They also engaged in outdoor activities with their family less often. In addition, being bullied was positively correlated to socioeconomic status and obese parents.

### Additional analysis

As the correlations presented in the above showed possible interrelationships between variables, additional multivariate analysis was performed to further explore whether variables were generated from a common underlying construct by means of linear regression and explanatory factor analysis. However, not all participants answered each question due to the crowdsourcing design (i.e., new questions could be created throughout the crowdsourcing process while members were not returning to answer those questions). A linear model using 10 imputed datasets containing all participants' answers to the first 15 questions showed that the four questions which were significantly associated to higher BMI’s were related to home environment (q4, parents’ obesity (β = 2.02, *p* = .011) and q5, living in poverty (β = 2.25, *p* = .018)) and diet and parenting style (q7, parents restricting child’s food intake (β = 2.48, *p* = .006) and q12, drinking juice/soda more often than water (β = .47, *p* = .009)). All four questions contributed to higher BMIs with positive coefficients. Hence, both food and non-food related questions were significant predictors of adult BMI in this model.

Additionally, exploratory factor analyses on the first 15 questions were performed using 2 to 4 factors. Different factor solutions were examined because the scree plot analysis indicated the inclusion of 2 factors whereas the Kaiser criterion suggested 6 factors. In each analysis, the food and non-food related questions grouped together while leaving out questions q2, q4, q7, q8 and q13. More specifically, questions q5 and q12 which emerged as significant predictors in the regression analysis loaded on different factors in each factor analysis. This means that the concepts which were significantly associated with a higher BMI (having obese parents (q4), parental restriction of food (q7), living in poverty (q5), and drinking juice/soda more than water (q12)) were not interrelated and measured by a similar underlying construct within the first 15 questions.

The first factor in each factor analysis had the largest weight on the question q3 (eating often at a fast food restaurant): the factor loadings on q3 for the 2 to 4 factor analyses were.52,.98, and.96, respectively. Moreover, the question q3 was not related to any other questions in the 3 and 4 factor analyses. Other non-food related interrelationships were also revealed in the 3 and 4 factor analyses. The question q5 (living in poverty) had a large weight (.52 and.59, respectively) and was grouped together with questions q9 (being involved in sports) and q11 (parents having a healthy relationship). Food related questions that grouped together in the 2 factor analysis were q6 (food used as a reward), q10 (eating late at night), q12 (drinking more juice/soda than water) and q15 (eating between meals). In the 3 and 4 factor analyses two questions remained grouped together: q6 (food used as a reward) and q12 (drinking more juice/soda than water).

The proportion of variation explained by the various factors was less than 8% for any individual factor. The chi-square goodness of fit test statistics improved when more factors were added (i.e., 2 factor model: *χ* ^2^ = 170.98, df =  89, *p* = 4.06e-07; 3 factor model *χ* ^2^ = 116.41, df =  75, *p* = .002; 4 factor model *χ* ^2^ =  91.11, df =  62, *p* = .01); however, the p-value did not exceed.05. This indicates that adult BMI is to be explained by additional and other constructs.

## Discussion

This paper explored the potential of crowdsourcing as a screening tool to evaluate whether the general public could identify early predictors that are associated with obesity development. Findings showed that participants were able to suggest various determinants that have been studied by professionals. However, some determinants that were extensively addressed by professionals were not associated with BMI among this sample. Most importantly, participants suggested potential predictors that are less well-documented in the literature, and that may suggest new directions for future research.

The questions which were created by the public through the crowdsourcing process covered numerous well-documented research areas. For example, although a well-known familial (or biological) factor of childhood obesity is parental weight [33], [38] which also came up in the crowd-suggested predictors, a more interesting finding is that one of the suggested questions was specifically about obesity of the maternal grandmother. This is possibly due to the fact that mothers were seen as the primary caregivers in the traditional families. In addition, the participants identified many other conventional predictors which are related to a healthy lifestyle such as specific topics related to dietary intake (e.g., milk, soda, snacking), physical activity (e.g., playing outdoors), hours of sleep, and television watching [11], [22], [28], [29]. Interestingly, two specific dimensions came up that might need more attention; that is, whether the family primarily prepared meals using fresh ingredients and whether children drank juice or soda instead of water. Although it has been shown that soda consumption is related to overweight [21], the specific way the question is asked by comparison to water drinking frequency might be more diagnostic.

In line with other recall studies of early markers for obesity [13], [14], questions concerning parental feeding style were associated to participant’s BMI. For example, using food to reward or punish behavior as well as a restrictive or controlling feeding style were associated with a higher BMI, however some related questions did not show significant associations. Other studies show that children whose parents engage in restrictive parent-child feeding practices (e.g., pressure to clean their plate) are more inclined to become overweight or obese [16], [40], [43] whereas a warm parenting style might be protective of health [19].

The positive influence of a supportive parenting style may be indicated by the lower BMI associated with having parents talk about nutrition and packing school lunches for their children. In addition, these two questions were related to other constructs that resembled a healthy lifestyle (e.g., use of fresh ingredients, outdoor activity with family, more sleep, drinking water rather than soda). It is possible that parents who talk about nutrition in an educational manner have a more positive impact on their children’s weight development than parents who talk about nutrition in the context of dieting and body image. Research has shown that mother being on a diet and maternal encouragement to be thin lead to a negative body image and restrained eating in young children [42]. In line with this tentative reasoning, it might be that parents who packed their children’s school lunch, talked about nutrition and were involved in family outdoor activities, practiced an involved, caring or supportive parenting style instead of a controlling style. Although school lunch participation and the healthiness of school lunches are currently under scrutiny [48], it appears that only one longitudinal study in the past has tracked school lunch participation and its association with obesity [49]. Hence, more research is needed to examine the underlying reasons of why parents pack children’s school lunches and whether there is a possible relation with BMI. Lunch packed by parents might be a protective factor for various reasons, possibly including supportive parenting style, healthiness of the lunch itself, and social environment at school, although it could also be indicative of socioeconomic status.

In line with what was mentioned in the above, people who had to prepare their own meals as a child more often than their parents did, had a higher BMI later on in life. Again, this question is likely to be related to a variety of other influential factors including parenting style, lifestyle and SES, as this question was related to poverty, less fresh vegetables in meal preparation, less family outdoor activities and less packed lunches to school. Speculatively, children whose parents were absent might have grown up in an unsupportive environment in which fresh produce was too expensive. Future research and intervention programs might profit from a more multidisciplinary approach by not focusing on either SES or parenting style but a combination as this might be related to a healthier lifestyle.

Apart from home environment, healthy lifestyle and family history, predictors of adult BMI were related to psychosocial well-being such as being bullied or having friends. Previous research has shown psychosocial and weight-related consequences of people’s social status; that is, bullying and peer rejection have been associated to a lower psychological well-being and a higher BMI [44], [50]. Longitudinal research is warranted to investigate whether adults became (or remained) overweight due to peer rejection during their childhood or whether they were rejected by peers due to their weight status at young age. 

Identification of interrelationships among conceptually related items was not done on the whole dataset due to the sparsity of the data. However, multivariate analyses performed on the first 15 questions resulted in groupings of questions that supported our own intuitive groupings in Table 3. For example, questions related to home environment naturally grouped together, but several questions also remained outside any of the factors. This suggests that although overarching themes were provided by the crowd through several interrelated questions, they also came up with independent concepts that might affect BMI. However, caution is warranted in interpreting these findings as they are based on only 27% of all questions. For a more comprehensive analysis (e.g., with more factors), improvement of crowdsourcing methodology is needed to ensure that most of the participants respond to all of the questions.

### Crowdsourcing: Involving the Citizen Scientist

The study demonstrated that crowdsourcing can be used to discover additional insights into obesity by taking advantage of the collective intuition and experience of the crowd, and is moreover a rapid method for collecting responses: experiment design, website deployment and data collection occurred in less than three weeks. In addition, crowdsourcing may have beneficial consequences for those who choose to participate: for example, showing participants which questions correlate with obesity could lead them to improve their parenting strategies, and get them involved in other citizen science initiatives to improve public health. Citizen science usually refers to engaging the public in large-scale data collection projects [51], which can be empowering and educational, and even motivate people to change their behaviors. The approach described here and in Bongard et al., [8] goes further by attempting to motivate subjects to couple their innate problem solving abilities with their own experiences with obesity. Another example of citizen science is the Quantified Self (http://quantifiedself.com), in which individual experimenters come up with novel ideas and hypotheses about factors influencing their health and behaviors [7]. Our approach however allows a group of participants to collectively discover determinants of healthy weight through indirect collaboration.

It is notable that only 7% of the participants in this study posed new questions. It would be interesting to examine what kind of people are the most enthusiastic and insightful citizen scientists in the context of obesity. One method of surveying participants’ motivations is described in [52], although the research domain is very different (engaging volunteers to classify galaxies). In further studies, the rationale for posed questions could be investigated by asking participants why they thought to ask that specific question. Some of the possible sources for ideas and hypotheses include personal experience, someone else’s experience, research, other literature, something that the person has seen or heard, just trying to think `outside the box’, or, perhaps most importantly, because of what other questions they saw on the site. This last motivator may help us to understand how certain questions, although not correlated with the health outcome of interest, nevertheless trigger another user to pose one that does correlate.

Crowdsourcing to generate research hypotheses and to screen obesogenic behaviors and factors is a relatively new approach. Hence, future studies would benefit from a checklist of questions to consider when setting up a crowdsourcing study. **Table 5** lines out a stepwise process for crowdsourcing from a social scientist’s perspective based on the lessons learned in this study and insights from related research.

**Table 5:** Leveraging Crowdsourcing for Research Insights.

Step	Considerations and relevant research	
Define the purpose of research	Define the outcome variable of interest	Success will depend on the ease with which participants can obtain accurate data for the outcome [8].
	Determine the level of crowd participation [55]	Contributory: provide data to researchers
		Collaboratory: assist in study development, data collection, and analysis
		Co-created: develop a study and get input from researchers
	Data collection	Observational, surveillance, or recall data among general population or specific target groups such as disease populations [56].
		Screening certain behaviors among certain target groups [8].
	Analysis and classification of existing data [52]	Crowd participation can enable handling huge datasets.
	Innovation	Generating new hypotheses [8].
		Creative solutions to problems [57].
		Creating content for interventions.
	Health education/Information sharing for collective benefit	Engaging the crowd to share ideas and support each other [58].
Determine the target group	Specific group or general public	General public can reliably perform simple tasks that everyone has some knowledge about.
		Knowledge-intensive tasks may be best accomplished by “nichesourcing”, gathering experts on a specific topic [59], such as a specific disease or condition [56].
Find the target group	Leveraging social media	Keyword approach to find relevant groups in Reddit.com, LinkedIn, Facebook, Quora, disease- or condition-specific networks (for example, www.TuDiabetes.org, a social network site for diabetics and their close ones), or even Craigslist.
		Keep in mind that people who are active in social media may be different from those who are less active, for example, in personality traits or need for cognition [60].
	General public, conventional channels	Most people have access to the Internet nowadays. Media coverage with a link to the website may attract a large number of participants (e.g.[52]).
	Pay participants: Amazon Mechanical Turk	Suitable for well-defined tasks [61].
Develop technology platform	Usability and attractiveness	Necessary and especially crucial with projects that wish to engage participants for a long time and/or get them to return to the website.
	Build or buy? [62]	Consider cost, development time, and technical proficiency that are required to develop the platform.
		Systems built from scratch are more flexible to modify, but require more development time.
		Ready-made platforms make it easier to focus on content, but they allow less freedom to modify the platform and functionality.
	Mobile in addition to/instead of web	Participants can passively share data that their smartphones sense (e.g. location, noise) or actively collect data (e.g. photos, surveys at certain situations, experience sampling) [63].
Attract the crowd	Make it simple, easy to participate, and valuable	Interesting, concise headline with clear, understandable message.
		Describe what the process will entail, and what the benefits from participating are.
		Allow various levels of participation. Some want to invest a lot of time, others perhaps only a couple of minutes.
		Participation will likely be greater if the site provides clear value (e.g., interesting insight into health outcomes)
	Make it easy to share	Let participants spread the word and make the study viral: Facebook, Twitter, email, repost links.
	Media coverage	Investigators can appear on television, radio, magazines, websites, and write in their blogs about the project [52].
Data collection and privacy	Privacy concerns	Certain types of data are more sensitive than others[64].
		People who are most willing to share their data and insights may be healthier and in better condition [56].
Compensation	What the crowd will get from participating?	Intrinsic motivation: altruism, advancing science, helping others, new knowledge about the outcome
		Researchers’ gratitude
		Credit for best performers, reputation
		Feedback for personal contributions
		Money
Involvement	How to keep participants engaged?	Answer questions and share findings
		Join conversations, be transparent
		Forum for participants to communicate with each other [52]
	Gamified systems to motivate	CitizenSort (http://citizensort.org/)
		Galaxy Zoo (http://www.galaxyzoo.org/) [52]

### Limitations and Future Research

Considerations need to be made when interpreting the findings of the present study. First, as new questions could be created throughout the crowdsourcing process, it was inevitable that not all participants answered each question. The first six questions gathered over 400 answers, whereas the last questions collected less than 100 answers. Due to the abundance of missing values, many questions were not able to be included in the multivariate analyses. Therefore, it was not possible to perform in-depth analysis to determine underlying and interrelated constructs. Future studies could greatly benefit from using an incentive which would motivate people to return to the site. This incentive would not necessarily need to be monetary; for some participants, intrinsic motivation to benefit science could be enough [7]; for others, an enjoyable game-like experience could be attractive [8], [53]. In addition, participants could be sent a reminder to return to the website after a few days.

Second, an appropriate sample size for analyses is difficult to calculate because the survey was voluntary and, moreover, we could not predetermine how many questions and answers the crowd would generate. As this study was exploratory in nature, we set a fixed time period of two weeks beforehand to find out how many participants we could attract in such a timeframe. The sample size we ended up with is comparable to prior crowdsourcing studies [8], [39] as 556 people participated in our survey within two weeks. An alternative approach in future research is to determine a target sample size beforehand and recruit until this sample size is reached.

Third, this was a retrospective study with self-reported responses about childhood experiences based on people’s recall. Therefore it is not possible to determine how the markers contributed to the development of people’s current BMI, and which adult behaviors and experiences might have caused weight changes. Furthermore, demographic variables were not controlled for in our study, and thus the validity of the findings in comparison to prior studies remains uncertain. Future studies should take demographic variables into account.

Fourth, the participants were recruited from online groups related to dieting and their BMIs might not have been stable. In addition, a sampling bias resulted from using these specific target groups. However, it is unknown whether dieters would pose different determinants for obesity than non-dieters. Therefore, this could have influenced the results in unknown ways; for example, certain associations between determinants and obesity may not have been captured because participants who answered those questions might have lost significant amount of weight already. Nevertheless, when it comes to weight loss or weight gain, nearly everyone has experience and is an expert. People who are interested in weight loss may have many diverse ideas regarding what may have led, personally, to weight gain or weight loss in their life; thus, they can be considered lay scientists in this field. The current study should be replicated, for example among a non-dieting sample, and participants should be asked about their highest lifetime weight to control for adulthood weight loss. Moreover, since participation was anonymous and non-incentivized, it is difficult to determine if responses were truthful or not. Some participants might have tried the system with different BMIs and varying answers just to see what would happen.

Fifth, the generalizability of the current findings may be limited. As the majority of participants were females in their late twenties, it is difficult to assess how the BMIs of males or seniors are influenced by the determinants. It would be interesting to investigate gender differences or whether there are differences between certain decades, for example concerning the impact of parenting styles. Nevertheless, crowdsourcing makes it relatively easy to assess determinants of behavior in subgroups which makes it a potentially beneficial approach to inform tailored interventions for specific target groups.

## Conclusions

This paper was one of the first to present crowdsourcing as a potential screening tool to evaluate whether the general public could suggest early predictors that are associated with obesity development. Findings show that participants were able to discover determinants that have been investigated by professionals. Most importantly, participants were able to highlight less well-documented topics which might need more attention in future research. However, some of the well-documented determinants from prior research were not found to be significantly associated with BMI in this study. These two observations highlight both the potential and the limitations of crowdsourcing. By engaging the general public in behavioral research, the crowdsourcing approach enables non-experts to proactively contribute insight to the research. However, because it is difficult to carefully control the quality of the questions submitted or the demographics of the participants, as would be the case with a more controlled study, this approach is most likely only a complement to, rather than a replacement for, conventional research methods. We suggest that insight generated from the crowdsourcing process can subsequently be used to develop new hypotheses, which could be tested in larger, more controlled longitudinal studies.

The potential new predictors discovered in this research were largely related to parenting styles and family environment. It would be worth investigating how parents could be taught to educate their children about food in a supportive manner as this ‘positive’ nutritional attitude might have an impact on their children’s eating habits and BMI later on in life. Looking at the general family lifestyle may provide broader explanations for the findings of this study. Given that engaging in outdoor activities with family, hours of sleep, and dietary patterns also emerged as significant correlates of BMI, healthy lifestyle during childhood in general is likely to be associated to a lower BMI later on. Habits learned and initiated in childhood tend to be continued in adult life, and therefore a stronger focus should be put on families as a supportive environment for establishing healthy habits [54].

This study also suggests several avenues for improving the crowdsourcing methodology. During this study, it became clear that the simple linear regression model used was not capturing all of the explainable variance in the BMI data. Future work will look at other ways to autonomously build models that better predict the outcome of interest. Better models will make it possible to give better feedback to participants about which questions impact predicted BMI (or other outcomes of interest). Experience with the crowdsourcing approach suggests that this feedback between the website and participants is an important motivator for participation. In future work we will study other ways to motivate participation, particularly ways to encourage participants to return to the site after their initial participation, or ways to find participants from more varied backgrounds.

## Supporting Information

Appendix S1 **The list of questions generated through crowdsourcing and their correlations with BMI.**
(DOC)
