# Supplementary material for: Crowdsourcing Novel Childhood Predictors of Adult Obesity
Source: PLoS One. 2014 Feb 5;9(2):e87756. doi: 10.1371/journal.pone.0087756 (PMC3914836; doi:10.1371/journal.pone.0087756)
Supplement: Appendix S1 — The list of questions generated through crowdsourcing and their correlations with BMI. (DOC) [file pone.0087756.s001.doc]

## Appendix S1. Questions generated through crowdsourcing and their correlations with BMI.

|  |  | **#** | **Question** | **Correlation** | **P value** | **n** | **Type** |
| --- | --- | --- | --- | --- | --- | --- | --- |
|  |  |  |  |  |  |  |  |
| **Home environment** | | | | | | | |
|  |  | | |  |  |  |  |
|  | **Food education** | | |  |  |  |  |
|  |  | 36 | When you were a child, you were taught how to cook. | .077 | .420 | 112 | no/yes |
|  |  | 59 | When you were a child...did your parents talk about nutrition? | -.309^**^ | .001 | 109 | no/yes |
|  |  |  |  |  |  |  |  |
|  | **Parenting / Parental feeding style** | | |  |  |  |  |
|  |  | 6 | When you were a child, were you rewarded with food? | .141^**^ | .005 | 403 | no/yes |
|  |  | 7 | When you were a child, did your parents restrict your food intake? | .155^**^ | .002 | 399 | no/yes |
|  |  | 13 |  |  |  |  |  |
|  |  | 19 | When you were a child... How many times per week did you bring your lunch to school? | -.234^*^ | .012 | 115 | numerical |
|  |  | 31 | Did your parents ask you what you were eating frequently? | -.151 | .102 | 119 | no/yes |
|  |  | 37 | When you were a child did your parents encourage you to clean your plate? | .071 | .453 | 113 | no/yes |
|  |  | 39 | When you were a child, was the food used as a punishment in any ways? | .219^*^ | .021 | 110 | no/yes |
|  |  | 40 | When you were a child, did your parents allow to eat whatever you wanted? | -.049 | .599 | 115 | disagree-agree |
|  |  | 45 | When you were a child, did your parents prohibit you eating certain foods? (e.g., sweets, sodas, etc) | -.062 | .514 | 112 | no/yes |
|  |  | 46 | When you were a child...highly sugary foods (candy, soda pop, etc) were a special treat rather than part of your regular diet. | -.073 | .462 | 104 | disagree-agree |
|  |  | 53 | When you were a child… did someone consistently pack a lunch for you to take to school? | -.345^***^ | <.001 | 106 | no/yes |
|  |  |  |  |  |  |  |  |
|  | **Parental dieting** | | |  |  |  |  |
|  |  | 21 | When you were a child... was your mother constantly on a diet? | .042 | .667 | 109 | disagree-agree |
|  |  | 25 | When you were a child... did your household serve reduced-fat alternatives to traditional foods (e.g. skim milk instead of whole, egg beaters instead of whole eggs, etc.)? | .161 | .091 | 111 | no/yes |
|  |  | 26 | When you were a child... were you aware of weight/body image being a topic of conversation or concern to the adults in your life (i.e. a parent dieting often or telling you to diet)? | .129 | .165 | 118 | disagree-agree |
|  |  | 38 | When you were a child how often did your parents exercise a week? | -.044 | .640 | 114 | numerical |
|  |  |  |  |  |  |  |  |
|  | **Household status and SES** | | |  |  |  |  |
|  |  | 5 | When you were a child, did you live in poverty? | .171^***^ | <.001 | 415 | no/yes |
|  |  | 11 | When you were a child, did your parents have a good healthy relationship? | -.043 | .408 | 367 | disagree- agree |
|  |  | 24 | When you were a child, were you raised by a single mother? | .081 | .397 | 112 | no/yes |
|  |  | 30 | When you were a child...did you usually eat together with your family? | -.075 | .423 | 115 | no/yes |
|  |  | 44 | When you were a child...did your parents divorce? | -.046 | .639 | 106 | no/yes |
|  |  |  |  |  |  |  |  |
| **Psychosocial well-being** | | | |  |  |  |  |
|  |  | 1 | When I was a child, I was bullied. | .128^**^ | .009 | 413 | disagree- agree |
|  |  | 20 | When you were a child...How many times a week were you left alone for longer than an hour? | .119 | .210 | 112 | numerical |
|  |  | 28 | When you were a child... did you experience any event that caused significant emotional trauma? | .116 | .219 | 114 | no/yes |
|  |  | 43 | When you were a child...Did you have many friends? | -.168 | .070 | 117 | no/yes |
|  |  | 48 | When you were a child... were you sexually abused? | .209^*^ | .026 | 113 | no/yes |
|  |  | 58 | When you were a child, were you facing identity issues that could have affected you psychologically? | .132 | .166 | 112 | no/yes |
|  |  |  |  |  |  |  |  |
| **Healthy lifestyle** | | | |  |  |  |  |
|  |  |  |  |  |  |  |  |
|  | **Diet** | | |  |  |  |  |
|  |  | 3 | When you were a child, how many times a week did you eat at a fast food restaurant? | .009 | .861 | 414 | numerical |
|  |  | 8 | When you were a child, how many times per week did you eat at a non-fast-food restaurant? | -.013 | .797 | 387 | numerical |
|  |  | 10 | When you were a child, did you often eat late at night? | -.003 | .947 | 380 | no/yes |
|  |  | 12 | When you were a child, did you drink juice or soda more often than water? | .166^**^ | .001 | 365 | disagree-agree |
|  |  | 15 | When you were a child, apart from breakfast, lunch, and dinner, how many times per day did you eat something in between meals? | .031 | .557 | 351 | numerical |
|  |  | 16 | When you were a child, how many times a week did you eat candy? | .063 | .476 | 129 | numerical |
|  |  | 23 | When you were a child... did you drink skim milk much more often than whole milk? | .107 | .268 | 109 | disagree-agree |
|  |  | 25 | When you were a child... did your household serve reduced-fat alternatives to traditional foods (e.g. skim milk instead of whole, egg beaters instead of whole eggs, etc.)? | .161 | .091 | 111 | no/yes |
|  |  | 27 | When you were a child... how many times a week did you eat sweetened cereal? | .065 | .497 | 113 | numerical |
|  |  | 29 | When you were a child... how often in a week would you eat home cooked meals? | .032 | .734 | 116 | numerical |
|  |  | 34 | When you were a child...did your family primarily prepare meals using fresh ingredients? | -.316^***^ | <.001 | 127 | no/yes |
|  |  | 52 | When you were a child... did your family grow their own food? | -.131 | .163 | 115 | disagree-agree |
|  |  |  |  |  |  |  |  |
|  | **Physical activity** | | |  |  |  |  |
|  |  | 2 | When you were a child, did you own a bike? | -.019 | .701 | 419 | no/yes |
|  |  | 9 | When you were a child, were you involved in any competitive sports? | .042 | .413 | 382 | no/yes |
|  |  | 17 | When you were a child did you engage in regular outdoor activity, like hiking or biking, with your family? | -.230^**^ | .008 | 130 | no/yes |
|  |  | 33 | When you were a child...catch and other active/outdoor games were your favorite. | -.143 | .120 | 119 | disagree-agree |
|  |  | 42 | When you were a child...How many hours per week did you play outdoors? | .030 | .757 | 112 | numerical |
|  |  | 51 | When you were a child, did you spend more time playing outdoors than indoors? | -.099 | .301 | 111 | no/yes |
|  |  |  |  |  |  |  |  |
|  | **Sleep** | | |  |  |  |  |
|  |  | 18 | When you were a child...How much sleep did you get on an average school weekday? | -.172^*^ | .034 | 152 | numerical |
|  |  |  |  |  |  |  |  |
|  | **Watching TV** | | |  |  |  |  |
|  |  | 50 | When you were a child...How times did you watch TV while eating dinner during the average week? | .016 | .869 | 108 | numerical |
|  |  | 56 | When you were a child... How many times a week did you have a meal while watching television? | .080 | .391 | 118 | numerical |
|  |  |  |  |  |  |  |  |
|  | **Dental care** | | |  |  |  |  |
|  |  | 47 | When you were a child... at what age was your first tooth filling? | .179 | .081 | 96 | numerical |
|  |  |  |  |  |  |  |  |
|  |  |  |  |  |  |  |  |
| **Built environment** | | | |  |  |  |  |
|  |  |  |  |  |  |  |  |
|  |  | 35 | When you were a child, were you raised on a coast of the United States? | -.021 | .819 | 120 | no/yes |
|  |  | 49 | When you were a child... Was a fast food restaurant within walking distance or a short bike ride? | -.007 | .944 | 115 | no/yes |
|  |  |  |  |  |  |  |  |
| **Family history & biological factors** | | | |  |  |  |  |
|  |  |  |  |  |  |  |  |
|  |  | 4 | When you were a child, were your parents obese? | .218^***^ | <.001 | 413 | no/yes |
|  |  | 14 | Were you born prematurely? | .039 | .468 | 356 | no/yes |
|  |  | 32 | What was your birth weight | .073 | .490 | 92 | numerical |
|  |  | 41 | When you were a child...were your grandparents overweight? | .198^*^ | .036 | 112 | no/yes |
|  |  | 54 | When you were a child...was your maternal grandmother obese | .208^*^ | .032 | 107 | no/yes |
|  |  | 55 | When you were a child...did you have any metabolic disorders | .057 | .527 | 125 | no/yes |
|  |  |  |  |  |  |  |  |
| * p <.05, ** p <.01, *** p <.001 | | | | | | | |
